# Metagenome-Assembled Genome of a *Cyclobacteriaceae* Bacterium, HetDA_MAG_MS6, Isolated from a *Trichodesmium* Consortium from Station ALOHA

**DOI:** 10.1128/mra.00454-22

**Published:** 2022-12-14

**Authors:** Joshua T. Roemer, Elaina D. Graham, John F. Heidelberg, Eric A. Webb

**Affiliations:** a Department of Biological Sciences, University of Southern California, Los Angeles, California, USA; b Department of Biological Sciences, Marine Environmental Biology Section, University of Southern California, Los Angeles, California, USA; Montana State University

## Abstract

Here, we describe the metagenome-assembled genome (MAG) HetDA_MAG_MS6. HetDA_MAG_MS6 was obtained from an enrichment of the heterocystous diazotroph HetDA, which was isolated near Station ALOHA. The MAG was placed in the *Cyclobacteriaceae* family and is predicted to be a chemoorganoheterotroph with the potential for ammonia uptake, phosphonate transport, and sulfolipid biosynthesis.

## ANNOUNCEMENT

*Trichodesmium*, a genus of nitrogen-fixing cyanobacteria, is present throughout the oligotrophic ocean and lives in association with multiple other organisms, including the diazotroph HetDA (1). The biogeochemical implications of the microenvironment in this consortium can be better understood by studying the genomes of each member (2).

Here, we describe a genome for a *Cyclobacteriacae* strain, HetDA_MAG_MS6. Initial samples were collected as detailed by Momper et al. (1). Briefly, *Trichodesmium* colonies were individually picked and transferred to sterile YBC-II medium (3) at 24°C with a 12-h light (100 μmol photons m^−2^ s^−1^)/12-h dark cycle. The enrichment was grown under the aforementioned conditions for 5 years prior to sequencing and was transferred to fresh medium every month. A 50-mL subsample of the enrichment was gravity filtered onto a 5.0-μm polycarbonate filter, and DNA was extracted using the Qiagen DNeasy PowerSoil kit. DNA was sent to the University of Southern California Epigenome Center and sequenced on an Illumina MiSeq sequencer (paired-end 250-bp reads, with a total of 4,725,335 raw reads) using the MiSeq reagent kit v2 with 300 cycles. Reads were trimmed using Trimmomatic v0.38 (parameters: –phred33, ILLUMINACLIP:TruSeq3-PE.fa:2:30:10 SLIDINGWINDOW:10:28 MINLEN:50) (4) and assembled using MetaSPAdes v3.14.0 (5). Coverage was calculated by mapping reads to the assembly with Bowtie2 v2.3.5 (6), and the reads were filtered using CoverM v0.4.0 (parameters: –min-read-percent-identity 0.95 –min-read-aligned-percent 0.75) (7). The assembly was binned using MetaBat2 v2.12.1 (8), BinSanity-wf v0.3.8 (9), and Concoct v1.1.0 (10) using default parameters, and DASTool v1.1. (11) was used to determine a final set of metagenome-assembled genomes (MAGs). Following this, all bins were run through MetaSanity v1.3.0 (12), PhyloSanity, and FuncSanity for phylogenetic placement and genome annotations.

CheckM (13) estimated that HetDA_MAG_MS6 was 95.66% complete, with 4,693,337 bp assembled into 406 contigs and an *N*_50_ value of 13,982 bp. The MAG had a GC content of 42.6% and a coding density of 92.4%. The MAG was determined to be unique via GTDB-tk relative evolutionary divergence (RED) scores (14, 15) and is in the family *Cyclobacteriaceae*, with the closest average nucleotide identity (ANI) matches falling below 80% at the family level.

In addition to a phosphate transport system (KEGG Orthology codes K02040 and K02036 to K02038), there are genes for phosphonate transport (KEGG Orthology codes K02044, K02041, and K02042). By harvesting phosphorus from the organophosphate compounds produced by cyanobacteria, this *Cylclobacteriaceae* strain may be better adapted to the oligotrophic ocean (16, 17). However, there is only a partial presence of the C-P lyase pathway used to cleave phosphorus from phosphonates (KEGG Orthology codes K02043, K06166, K06167, K06162 to K06164, K05780, K05781, K05775, and K09994) and no indication of alternative cleavage pathways (18).

HetDA_MAG_MS6 also has a putative pathway for sulfolipid biosynthesis (KEGG Orthology code K06118), which may allow it to reduce further its phosphorus demand (19). While heterotrophic bacteria do not utilize sulfolipids as commonly as cyanobacteria do, heterotrophs living in phosphorus-limited environments can still possess and express genes for sulfolipid biosynthesis, as the pressure of conserving phosphorus outweighs the pressure of genome streamlining (20).

In summary, HetDA_MAG_MS6 contains genes for ammonia uptake, phosphonate transport/utilization, and sulfolipid biosynthesis. These genes allow the bacterium to survive in its oligotrophic environment in association with the *Trichodesmium* consortium.

### Data availability.

Raw sequences and genomes are available under BioProject accession number PRJNA719568. Raw reads were deposited in the SRA under accession number SRR14140256. The genome is available under BioSample accession number SAMN18613314.
